# *Punica protopunica* Balf., the Forgotten Sister of the Common Pomegranate (*Punica granatum* L.): Features and Medicinal Properties—A Review

**DOI:** 10.3390/plants9091214

**Published:** 2020-09-16

**Authors:** José Antonio Guerrero-Solano, Osmar Antonio Jaramillo-Morales, Tania Jiménez-Cabrera, Thania Alejandra Urrutia-Hernández, Alejandro Chehue-Romero, Elena G. Olvera-Hernández, Mirandeli Bautista

**Affiliations:** 1 Academic Area of Pharmacy, Institute of Health Sciences, Autonomous University of the State of Hidalgo, San Agustin Tlaxiaca, Hidalgo 42160, Mexico; jose_guerrero@uaeh.edu.mx (J.A.G.-S.); tania_jimenez@uaeh.edu.mx (T.J.-C.); chehuea@uaeh.edu.mx (A.C.-R.); olverae@uaeh.edu.mx (E.G.O.-H.); 2 Department of Nursing and Obstetrics, Division of Life Sciences, University of Guanajuato, Ex Hacienda el Copal Km 9 Irapuato-Silao highway ap 311, Irapuato, Guanajuato 36500, Mexico; 3 Academic Area of Food Chemistry, Institute of Basic Sciences and Engineering, Autonomous University of the State of Hidalgo, Pachuca-Tulancingo km 4.5 Carboneras, Pachuca de Soto, Hidalgo 42184, Mexico; thania_urrutia9356@uaeh.edu.mx

**Keywords:** *Punica protopunica* Balf., *Punica granatum* L., *Punica* genera, *Lythraceae*

## Abstract

*Punica protopunica* Balf. is one of only two species housed by the *Punica* genera. *Punica protopunica.* Balf., known as Socotran pomegranate, is an endemic, isolated species found only in Socotra archipelago in the northwestern Indian Ocean, and is considered to be the ancestor of pomegranate. This review stems from the fact that in many *Punica granatum* L. articles, *Punica protopunica* Balf. is mentioned, but just in an informative way, without mentioning their taxonomic and genetic relationship and their medicinal properties. It is there where the need arises to know more about this forgotten species: “the other pomegranate tree.” A large part of the human population does not know of its existence, since only its “sister” has spread throughout the world. The present review deals with the taxonomy and origin of *Punica protopunica* Balf., the morphology of the tree, distribution, cultivation, vulnerability, and as well as its relationship with *Punica granatum* L. It also discusses its uses in traditional medicine, its antioxidant capacity, and the medicinal properties of this forgotten species.

## 1. Introduction

Myrtales is an order within the classification of terrestrial green plants (Viridiplantae-Streptophyta) [1,2]. The Lythraceae family (from the order of the Myrtales) is composed of herbs, shrubs and trees that are mainly recognized for their flaky bark, crumpled petals from the bud (when emerging out of the rim of the calyx tube of the sepals), leaves oppositely paired, seeds with multi-layered outer integuments, and the fruit is usually a capsule [1,2,3,4]. The Lythraceae family comprises 31 genera including the *Punica* genera [5]. This is quite surprising as the *Punica* genera has previously been assigned to the monogenic *Punicaceae* family [6,7]. However, the results of numerous molecular and morphological investigations revealed the close relationship of the genera *Punica* with the Lythraceae family [1,8,9,10,11,12,13]. Previously it was considered a monogenic Punicaceae family that contains only one genera, *Punica* [14,15]. According to Pliny, the name *Punica* was given by the Romans, referring to the city of Cartago, in Tunis (Punic, Phoenician, Carthaginian), from where the best pomegranate (from Latin “*pome*” witch means apple and “*granate*” meaning many seeded) arrived in Europe. The genera *Punica* contains two species, *Punica granatum* L. and *Punica protopunica* Balf., [16]. Initially, *Punica granatum* L. was known as *Malum punicum*, the apple of Cartago, but later, Carl Linnaeus (1707–1778) chose the current name, with a specific epithet of *granatum*, which means granular [6]. On the other hand, *Punica protopunica* Balf. was first described by the Scottish botanist Isaac Bayley Balfour (1853–1922) during his arboreal and botanical expedition in 1880, and published in the Proceedings of the Royal Society of Edinburgh in 1882 [4,14,15,16]. *P. granatum* is native to the region that covers territories from a part of Iran to northern India [17,18]. Wild *P. granatum* L. types have their natural distribution in central Asia from Iran, Afghanistan, Turkmenistan, to northern India, and this region is considered the center of origin of pomegranate [6]. Later, the pomegranate was distributed to the Mediterranean, East Asia, America and South Africa, and this distribution originated the genetic diversity of the pomegranate [19], on the other hand, *P. protopunica* Balf. is endemic to the Socotra archipelago (located between the Arabian sea and the Guardafui channel in the Indian Ocean, off the coast of the Horn of Africa) [20]. In this context, the objective of this review was to compile the available information on the *P. protopunica* Balf. species (morphology, distribution, cultivation, vulnerability, uses), including its antioxidant capacity and the medicinal properties, to make it known and allow a wider use of this forgotten wild species.

## 2. Results

### 2.1. Taxonomic Positioning and Distribution

*P. protopunica* Balf. (taxonomic positioning in Table 1) is an endemic species, found only in the remote archipelago of Socotra, and is considered as one of the most important endemic species on the archipelago [21,22]. Socotra belongs to the Republic of Yemen, it is located at 12°19’–12°42’ N and 53°18’–54°32’ E, on the Arabian sea of the Indian Ocean [21,23]. Socotra archipelago, also known as the “Galápagos of the Indian ocean” is a group of four islands, Socotra being the most important and largest one. Socotra archipelago (isolated from the rest of the world) is an island with great biological diversity (approximately 900 plant species, 30% endemic) and it hosts unique fauna and flora [24]. People of Socotra use medicinal plants and it is known that this people has a deep respect for nature and its environment [24]. Socotra was included in 2008 to the select list of World Heritage by the United Nations Educational, Scientific and Cultural Organization (UNESCO) under the criteria of natural site [25]. Additionally, *P. protopunica* Balf. is considered from an independent evolutionary path (due to isolation from the rest of the world) [26], commonly considered as “the other pomegranate tree”, it is an unknown species worldwide, in contrast to the sacred status granted by the name of its species, *protopunica*: prototype [27].

*P. protopunica* Balf. is distributed in different regions of the island, but mainly grows in the central-western part of Socotra, in humid forest regions, in the Haggeher mountains whose slopes are characterized by being made of granite and having a maximum elevation of 1503 m. It also grows on Diksam, the island’s limestone plateau, which rises precipitously 1520 m above sea level. The total area covered has been calculated to be 1/15 of the total area of Socotra (3796 km^2^) [27].

### 2.2. Morphology

Balfour, the discoverer of *P. protopunica* Balf., described it as “trees with branches, often thorny; elliptic leaves round sheath, oblique; below the oblong, obtuse flower bracts; obovate petals; joined carpels, horizontal basal tone center spiral. From Socotra, a new species that abounds and grows on the peninsula” [30]. Additionally, Balfour wrote: “In general habit, it is not unlike the pomegranate, but its leaves are larger and coarser, and it wants the delicate character of the foliage of that species. The flowers, too, are somewhat smaller, and their turbinate base is more angular. The fruit is very much less in size” [31]. *P. protopunica* Balf. has morphological differences compared to *P. granatum* L.; it has larger, narrower leaves, different foliage, continuous flowering, and smaller, pink (not red) flowers. The fruit of *P. protopunica* Balf. is round, pommel-shaped, with a maximum diameter of 3 cm and a characteristic yellow-greenish or red-brown color when ripe, is smaller, evergreen, with white seeds and less sweet than *Punica granatum* L. [20,30,31,32,33]. Table 2 shows morphological characteristics of the species.

### 2.3. Cultivation

A hardiness zone is a geographical area defined in the quality of a specific category of plant life capable of growing. The most widely used system is that of the United States Department of Agriculture (USDA), which includes 13 zones characterized by annual extreme minimum temperatures. The use of this system has spread throughout the world and has been adapted in other countries. Using this system, *P. protopunica* Balf. has USDA hardiness zone 7a through 11b; from −17.8 °C to +10 °C [20], which means that, emulating optimal conditions, it can be grown in other regions of the world.

*P. protopunica* Balf. can grow out of the island of Socotra, however certain requirements must be met that are necessary for its growth: (1) temperatures above 10 °C; (2) sunlight from 6 to 8 h per day and protection against the wind; (3) constant accumulation of humidity (1000 mm) with a percentage of 20% to 40%; and (4) alkaline soils with pH 7 and content of calcareous or rocky gravel. In cultivation, sowing, cutting and grafting can be methods of propagation. Botanist Alan Radcliffe-Smith from the Kew Royal Botanic Gardens Herbarium (1938–2007) successfully propagated *P. protopunica* Balf. using all three methods, although cutting and grafting did not result in fruit in the varieties [22,27].

### 2.4. Vulnerability and Conservation of the Species

As early as 1978, *P. protopunica* Balfwas considered a vulnerable species with fragmented populations. Later in 2004 [34] the red list of the International Union for Conservation of Nature (IUCN) specified that *P. protopunica* Balf. was a vulnerable species; It indicated that efforts should be made to protect *P. protopunica* Balf., since it is the only congener of *P. granatum* L., although, according to Miller [29,34], this information is outdated, since it has been shown that *P. protopunica* Balf. is widely found on the island of Socotra and is quite common in some regions. Its total area of occupation is approximately 100 km^2^, 2/15 of the total area of Socotra. Miller also reported that *P. protopunica* Balf. has a fragmented distribution with different subpopulations and regenerates actively, however, there are large areas in which the tree does not grow, except in some areas with remaining populations without regeneration being observed. The foliage of *P. protopunica* Balf. is of no interest to livestock and the tree is not cut down for fuelwood, even in dry spells. Wood is not important neither as firewood nor for construction.

Socotra was included on the World Heritage List in 2008 as a natural site, which has had a positive impact on the conservation of its species [35]. The inclusion was under the selection criteria number 10 that establishes: “to contain the most important and significant natural habitats for in-situ conservation of biological diversity, including those containing threatened species of outstanding universal value from the point of view of science or conservation”. In its operational guidelines, UNESCO requests that in order to maintain this status, the assets and properties included on the list must be protected by well-established and delimited legislation and institutional regulations, to guarantee their safeguarding. Likewise, the states must demonstrate that actions are carried out to protect them at the national, regional and local levels, and must attach the appropriate texts to the nomination with a detailed explanation of how this protection operates [36]. So the archipelago species, including *P. protopunica* Balf., are now more protected than ever, however, UNESCO has detected some factors that affect the property, such as livestock farming/grazing of domesticated animals, management plan, uncontrolled developments including ground transport infrastructure: the road network, absence of biosecurity politics to eradicate the introduction of invasive species, extreme weather events (storms and cyclones) and industrial activities among others [37,38]. There is a development and conservation program for the archipelago called: Socotra Archipelago Conservation and Development Program (SCDP). This is an initiative of the Republic of Yemen to develop and conserve the archipelago’s resources. The SCDP is supported by the United Nations Development Program (UNDP) and the governments of three European countries (Italy, Poland and the Netherlands), in conjunction with international donors and private non-profit organizations. The mission of the SCDP is to join the efforts of all the aforementioned organizations and countries for the human development of the population and conservation of the biodiversity of the Socotra archipelago with a sustainable approach [39].

### 2.5. Relationship between P. protopunica Balf., and P. granatum L.

In 1973, Shilkina reported that Socotran pomegranate tree wood contains fiber tracheids, (water-conducting cells). She found that tracheids are the only characteristic that differentiates *P. protopunica* Balf. from the rest of botanical and arboreal varieties of the order Myrtales. She stated that this unique characteristic is not shared by even *P. granatum* L. Therefore, she urged that *P. protopunica* Balf. be not only the only member of its species, but also the only species of a new genera. It appears that, based on the anatomy of the xylem, *P. protopunica* Balf. was suggested as an ancestor of the domesticated species *P. granatum* [7]. On the other hand, in *P. protopunica* Balf., 2n = 14, therefore, the haploid number of chromosomes is n = 7, unlike n = 8 in *P. granatum* L.; this difference is considered as a primitive characteristic of *P. protopunica* Balf. from the evolutionary point of view, since n = 8 is a development factor [40].

Recently, Youssef et al., [33] analyzed genetic diversity and the relationship between *P. protopunica* Balf. and eleven accessions of *P. granatum* L. (from Egypt, México and Yemen), using the following molecular markers: (1) amplified sequence-related polymorphism (SRAP); (2) amplification polymorphism of the target region (TRAP); (3) amplified intron-directed polymorphism (ITAP); and (4) sequence analysis of the *pgWD40* gene (involved in anthocyanin biosynthesis). It was found that the relationship between accessions of *P. granatum*, grouped by regions, was approximately 90% similar, while, evidently, the degree of genetic variation was altered within of each region. However, these markers revealed the relationship between *P. protopunica* and *P. granatum* at 33% similarity. ITAP, TRAP and SRAP generated a total of 719 bands, of these, 193 were specific for *P. protopunica* Balf., and 234 bands were shared between both species. The pgWD40 gene analysis showed 100% identity between *P. granatum* L. accessions and 98% with *P. protopunica* Balf. Phylogenetic analysis of the WD40 sequences of species, including both species of the *Punica* genera confirmed the relationship between *P. protopunica* Balf. and *P. granatum* L., supporting the hypothesis that *P. protopunica* Balf. could be an ancestor of *P granatum* L.

Moreover, Muhammad et al., [41] studied the genetic association among the genotypes of the species of *Punica* (20 genotypes of *P. granatum* L., 20 of *P. protopunica* Balf.), based on morphological and biochemical characterization. The phylogenetic tree was constructed with the 40 genotype data matrix based on morphology to represent the similarity of the two species. The phylogenetic tree divided the two species in two lineages (Regions R-I and R-II). R-I holds the 20 genotypes of *P. granatum* L., while R-II enclosed all genotypes of *P. protopunica* Balf. The similarity indexes were performed for the genotype of 2 species that was 53.84% for *P. protopunica* Balf. and *P. granatum* L. In the biochemical characterization, the total seed protein profiling was carried out on slab gel electrophoresis; 10 bands were recorded in both species (molecular weight 15 KDa–180 KDa) intra locus contribution toward the genetic disagreement was 10% in *P. protopunica* Balf. and 50% in *P. granatum* L. Inter species locus contribution toward genetic diversity was 50%.

### 2.6. Uses in Traditional Medicine

Despite the great scientific advancement in medicine and pharmaceuticals, much of Yemenis actively practice traditional medicine, they use medicinal plants for their daily health care needs and have a long tradition in herbal medicine. The vegetation and flora of Socotra provide healers with “natural pharmacies” with a great variety of plants, to prepare phytomedicine, in order to alleviate a great variety of human and veterinary diseases [42]. Some authors have reported the use of *P. protopunica* Balf. fruit peel, seed and flower in traditional medicine; the extraction and consumption techniques are by decoction, boiling, infusion, maceration of ethanol and fresh juice. The above mentioned are helpful for treating diseases such as peptic ulcer, diarrhea, dysentery, sores and wounds, urinary infections, dry cough, digestive problems, skin disease, mouth and throat sore and jaundice. It is also used because of the anthelmintic and anti-diabetic properties [29,34,41,43,44,45,46].

### 2.7. Bioactive Compounds of Pomegranate and Their Medicinal Properties

#### 2.7.1. Phenolic Content and Antioxidant Activity

Muhammad et al., in 2019 [41], evaluated the antioxidant potential of methanolic extracts of *P. protopunica* Balf. and *P. granatum* L. species, both cultivated in Swat Valley, KP, Pakistan. They found high amounts of total phenols in both species, as well as flavonoids and antioxidant activity, with *P. prototunica* Balf. showing the highest flavonoid content. Antioxidant activity was similar between species. The other study was led by Al-Huqail et al., 2018 [47]. They studied the antioxidant effect of the aqueous ethanolic extracts of the peel and seed coat of *P. granatum* L. and *P. protopunica* Balf., in vitro. The two extracts not only contained significantly different phenolic and total flavonoid contents but also different phytochemical constituents. Gas chromatography mass spectroscopy (GC-MS) analysis of the peel extracts revealed twenty-six compounds. The main ones were benzenepropanoic acid, 1H-pyrrole-2,5-dione, 1,2-benzenedicarboxylic acid, 1-(propylthio)-(CAS) ethanol (CAS) ethylalcohol methyl ester of 3-methoxypropionic and 2-propanol. *P. protopunica* Balf. seed coat extract showed the presence of 14 phytochemical constituents, the major constituents were Di-2 (2-ethylhexyl) phthalate, 1,2-benzenedicarboxylic acid, propanoic acid, 2-hydroxy-ethyl formic acid and benzoic acid. In the malondialdehyde method (MDA), hydrogen peroxide (H_2_O_2_) scavenging and DPPH· assays, the two seed coat extracts exhibited very high antioxidant activities, with higher activity observed for the *P. granatum* L. extract [47]. These differences in the antioxidant activity in the two species may be attributed to their different phytochemical constituents. The importance of the high concentration of phenolic compounds is that they protect cells from the damaging effect of free radicals, molecules responsible for altering biological systems, causing diseases or accelerating aging [48].

#### 2.7.2. Antimicrobial, Antiviral and Antiprotozoal Activity

Mothana and Lindequist [49] evaluated the antimicrobial effect of twenty-five medicinal plants of the island of Socotra, including the fruit and leaves extracts of *P. protopunica* Balf. (4 mg of the dried extract), on nine types of Gram-positive and Gram-negative bacteria: *Bacillus subtilis* (ATCC 6059), *Candida maltosa* (SBUG), *Escherichia coli* (ATCC 11229), *Micrococcus flavus* (SBUG 16), *Pseudomonas aeruginosa* (ATCC 27853), *Staphylococcus aureus* (ATCC 6538), multi-resistant strains *Staphylococcus aureus*, *Staphylococcus epidermidis* 847, and *Staphylococcus haemolyticus* 535 and against a species of yeast. The methanolic extract of *P. protopunica* Balf., was found to be one of the species with the highest antimicrobial activity, especially on Gram-positive bacteria including multi-resistant strains of *Staphylococcus*, but without activity on yeast.

The antiviral activity of the methanolic and aqueous extracts of twenty-five medicinal plants including *P. protopunica* Balf. fruit and leaves, were evaluated in two in vitro models (unreported concentrations) by Mothana et al., [50], one with MDCK cells with type A influenza virus and the other with Vero cells infected with herpes simplex virus type 1 (HSV-1). HSV-1 was more sensitive than type A influenza against the extracts evaluated. The half maximal inhibitory concentration (IC_50_) for *P. protopunica* Balf. was anti-influenza virus A = 75.7 µg/mL and anti-HSV-1 = 5.8 µg/mL. The species was not considered by the authors as a plant with potential for the development of antiviral drugs.

Additionally, Mothana et al., [45] evaluated the in vitro antiprotozoal activity of twenty Socotra plants including the methanolic extract of the fruit of *P. protopunica* Balf., (at 5 concentrations: 0.25, 1, 4, 16, and 64 µg/mL), *Plasmodium falciparum* erythrocytic schizonts were used to evaluate antiplasmodial activity. The antileishmanial activity was evaluated using a model of intracellular amastigotes of *Leishmania infantum*, and finally the antitripanosomal activity was evaluated using intracellular amastigotes of *Trypanosoma cruzi* and free trypomastigotes of *T. brucei*. The results indicated that there is selective activity of *P. protopunica* Balf., against *Plasmodium* (IC50 2.2 μg/mL), and a potential for relevant antileishmanial and antitrypanosomal activity was also found. In the same line of research, Barzinji et al. [44] investigated the antimalarial efficacy in vitro of methanolic and aqueous extracts of thirteen traditionally used plant species from Yemen, including *P. protopunica* Balf., in blood samples from positive malaria patients. The methanolic extract from *P. protopunica* Balf. (20 mg) was one of the three extracts with the highest antimalarial activity (IC 0.98 µg/mL), and also exhibited schizont maturation inhibition (SMI) of 31.25 μg/mL [49].

#### 2.7.3. Anticancer Activity

In 2007, Mothana et al., published the study “anticancer potential of Yemeni plants used in folk medicine”. Twenty-four methanolic extracts of common plants in traditional medicine in Socotra and other Yemen states were evaluated. *P. protopunica* Balf., (leaf and fruit extracts) was included. They used a microtiter plate assay based on cell staining with violet crystal to evaluate the in vitro cytotoxic potency of the extracts at different concentrations, with 5 human cancer cell lines: two urinary bladder carcinomas (5637 and RT-112), two lung cancer line (A-427 and LCLC-103H) and a breast cancer line (MCF-7). The methanolic extracts of *P. protopunica* Balf. exhibited a moderate potency of toxicity (seventh place of the extracts evaluated) in all tumor cell lines with IC_50_ values of 16.5 to 37.6 µg/mL. The methanolic extract of *Dendrosicyos socotrana* had the greatest cytotoxic effect against all the cancer cell lines analyzed [51].

#### 2.7.4. Cytotoxicity

In some of the studies cited above, cytotoxicity tests were carried out. Cultured MRC-5 SV2 cells were used for the toxicity test for *P. protopunica* Balf. fruit and leaves extracts. Cell viability was evaluated fluorimetrically. Fluorescence was measured and cell viability results were compared versus control group (data were expressed as a percentage reduction in cell viability). The IC_50_ for *P. protopunica* Balf. was 29.5 ± 3.7 µg, which is interpreted as low toxicity by the authors [45]. A cytotoxicity assay on the proliferation of MDCK and Vero cells was also carried out using culture plates and incubated at 37 °C with 5% CO_2_. Confluent monolayers were incubated with dilutions of extracts (100, 50, 25, and 12.5 µg/mL) in culture medium for 3 days. The 50% inhibitory cellular concentration (ICC_50_) was measured by a dye absorption assay, with culture medium as a control. The ICC_50_ of the pomegranate was less than 10 µg, which was considered non-cytotoxic [50]. With the little information available, it cannot be stated that there is no toxicity of the tree and its components, however the trials point to that conclusion.

## 3. Discussion

Socotra Archipelago is known to be a section of what was once the Gondwana Supercontinent [52,53]. The biodiversity and geology of the island of Socotra are living and tangible proof of the historical biogeography of the supercontinent of Gondwana [54]. In the early Cretaceous, Gondwana included the continental areas of Africa, South Asia, Polynesia and Central America, which later separated to create the continents as we know them today. Subsequently, the 4 islands of the Socotra archipelago, was isolated from the Indian Ocean approximately 20 million years ago, when the African and Arab plates separated, which resulted in the formation of the Gulf of Aden [52,55]. At some point in that transformation, the Socotra archipelago was left behind, isolating all the living matter contained in that piece of land. It is known that Socotra is not an island formed by volcanic lava but is a separate portion of a continent, so the endemic species are really old and “rare” in the eyes of modern humans. The dragon’s blood tree *Dracaena cinnabari* Balf. and Socotran *Adenium obesum* tree are clear examples, that look like they came out of a science-fiction novel. This is how many of the species that have been home to the island for millions of years had a different evolution, with fewer changes than in other lands, hence the genetics of *P. protopunica* Balf.

The morphological differences between *P. protopunica* and *P. granatum* are evident, but the most notable are the size of the tree, which can be more than 7 m in natural conditions for *P. granatum* L. [41], as opposed to the 4.5 m (average height) for *P. protopunica* Balf. [20]. The flowers are orange to red in *P. granatum* L. and smaller bright pink in *P. protopunica* Balf. [27,56], and the color of the fruit peel in *P. granatum* L. can be yellow, reddish yellow, or different shades of green and red (starting from pink to crimson); purple is less common and there are even unique varieties, such as black pomegranate, which acquires its coloration from immaturity and remains so until overripe [40,57]. On the other hand, as we already mentioned, *P. protopunica* Balf., has a fruit that ripens from green to greenish yellow, which can have a dark pink hue and they are in continuous bloom [23,29].

As we have seen, *P. protopunica* Balf. is endemic to Socotra and the distribution is centered on the island, however one of the studies we reviewed used specimens of *P. protopunica* Balf. from Pakistan (it was introduced to this region) [41]. It has been reported that with special care, the species can be cultivated outside the region where it grows naturally [22], making it a viable option for the species to be cultivated in other regions of the world, and taking into account vulnerability reports in Socotra and thus preserve it in a better way. In addition, efforts have been made to collect, conserve and evaluate the germplasm of *Punica* species [58]. There are collections of germplasm of wild and domesticated varieties of pomegranate in gene banks and seed banks of Albania, China, Cyprus, Egypt, France, Germany, Greece, Hungary, India, Iran, Israel, Italy, Morocco, Portugal, Spain, Tunisia, Turkey, Turkmenistan, Ukraine, USA and Uzbekistan [59,60,61]. However the largest collection is located in St. Petersburg, Russia. Interestingly, there was a collection considered the largest, in Garrygala Turkmenistan, but Levin reported that it was destroyed when Turkmenistan separated from Russia [40].

The domestication process of *P. granatum* L. gave rise to fruits and plants with magnum seeds, some infertile seeds and fruits, as well as fruits and seeds of different shades of color [58]. Chandra et al. (2010) [19] gave us a detailed history of the pomegranate, explaining that this was from the first fruit crops to be domesticated and planted in the years 4000 and 3000 BC., being one of the oldest edible first fruits [19,62]. It is known to have been cultivated in Egypt and consumed in India (it was an important food in Indian royalty) so early that there is a Sanskrit word for pomegranate. There are also records of its consumption in China during the Han and Sung dynasties, carried from the Middle East by merchants. It was adopted and consumed regularly in medieval Europe and spread around the world in European conquests [60].

Due to the globalization of pomegranate cultivation, there are genetic variations within the same species. More than 500 varieties of *P. granatum* L. are known, although few varieties bring their cultivation to a commercial level of production (about 50) [63]. In contrast, *P. protopunica* Balf. has a smaller wild fruit, a lesser variety of colors, and an acidic flavor that makes it an inedible fruit. There is a great polarization when referring to the two species of the *Punica* genera, on the one hand, there is the common pomegranate, widespread throughout the world, reported as a super fruit in any recipe magazine article (*P. granatum* L.). The other is the Socotran pomegranate (*P. protopunica* Balf.), which is little known, hidden from the eyes of the world and only used by the people of Socotra for medicinal purposes, the one without admirers.

*P. protopunica* Balf. is one of the only two species of the *Punica* genera, being considered as the “sister” of *P. granatum* L. [14]. However, according to its origin (due to its independent evolutionary line and which seems to be an ancestor of the *Punica* genera), we think that it may be, beyond the taxonomic classification, the “grandmother” of *P. granatum* L.

*P. protopunica* Balf. has strong ties to *P. granatum* L., and a strong relationship with the flora of the adjacent continental areas of Arabia and Northeast Africa, tropical Africa, Madagascar, India, South Asia, Polynesia and Central America that, as already mentioned, were united in the Cretaceous. It is believed that at least since the late Cretaceous, much of Socotra was emerged, considering itself one of the longest isolated landmasses on earth. Its vascular plants have an endemicity index, quite similar to that of other islands such as the Canary Islands, and it is a refuge for interesting paleoendemisms of very ancient origin, including the case of *P. protopunica* Balf. [64].

Herbalism is one of the most used treatments in traditional Yemeni medicine, predominately in rural territories, herbalism is practiced by Yemen population to all kind of ailments [65]. The uses of *P. protopunica* Balf. in traditional medicine agree with the uses that have been reported in *P. granatum* L., although *P. granatum* L. has more uses and has had more effects attributed to it. *P. granatum* L. is traditionally used for diarrhea, stomatitis, ulcers, bleeding, enemas, vaginal discharge, inflammation of the pancreas, gallbladder diseases, dysentery and stomach disorders, antiparasitic (taenicide and others), antibacterial, inflammatory diseases, astringent, abortion, burns, pain, snakebite, bronchitis, cough, and nausea [66,67,68,69,70]. Traditional and alternative medicine has many followers in the Republic of Yemen, because access to occidental medicine is still restricted, and Yemen is a country with difficult access [71].

Pomegranate is a source of bioactive compounds, present both in the fruit (peel and arils), and in the leaves and bark [72]. Ozgen et al. [73] affirm that pomegranate is a fruit rich in phenolic antioxidants, specifically anthocyanins, but their content varies between varieties, or sub-species. The fruit has a large number of flavonoids, it is estimated that about 0.2% to 1% of the weight of the fruit represent this group of compounds, of which about 30% of all the anthocyanidins are in the peel [74]. Pomegranate juice has a high content of polyphenols, significant amounts of ellagic acid, caffeic acid, chlorogenic acid, coumaric acid, catechins, ferulic acid and a large list of anthocyanins [75]. The literature indicates that pomegranate contains 124 different compounds and that among these phytochemicals, high molecular weight polyphenols (such as ellagitannins and punicalagin) are likely to mediate most of the fruit’s protective effects against harmful agents [75]. In the peel, almost 48 phenolic compounds have been identified [76]. Some authors identified and quantified phenolic compounds with more than 50 varieties of pomegranate fruits, using high-performance liquid chromatography with electrospray ionization and mass spectrometry (HPLC-DAD-ESI/MS^n^), they concluded that ellagitannins were the most abundant compounds in all the investigated samples (mesocarp, peel, arils, juices), and all the varieties had ellagitannins and anthocyanins [77,78]. All these data were taken from studies in *P. granatum* L. It can be hypothesized that these compounds are also present in *P. protopunica* Balf., however there is a large information gap in this regard. Hundreds of studies have been conducted in which the antioxidant capacity and total phenols of *P. granatum* L. are evaluated, however, for *P. protopunica* Balf., the scenario is different. It is curious that there are not enough published studies of the content of total phenols and antioxidant effect of *P. protopunica* Balf. Nor did we find studies of the nutrimental and chemical composition of the fruit. This may be due in the first place to the isolation of the species, and also due to the difficulty in obtaining government permits, as it originates from a declared a World Heritage Site. Perhaps there are unpublished works.

Many of the uses in traditional medicine have been explained and demonstrated with a great diversity of in vitro and in vivo studies. For example, in *P. granatum* L. hypoglycemic, hypocholesterolemic, hypotriglyceridemic, antihypertensive, anti-atherosclerotic, anti-inflammatory, against metabolic syndrome, various types of cancer, antimicrobial and antifungal, healing, among others effects were reported [79,80,81,82,83,84,85,86,87,88,89,90,91,92,93,94]. However, not enough reports of biological effects of the *P. protopunica* Balf., species were found. The antimicrobial, antiviral, antiparasitic and anticancer activity found in *P. protopunica* Balf. has also been found in *P. granatum* L., and many of the mechanisms of action reported by the authors are related with the high concentration of bioactive compounds of secondary metabolism of the fruit, specially ellagic acid, gallic acid, punicic acid and flavonoids as quercetin and kaempferol [45,77,82,85,87,94,95,96,97,98,99,100,101,102]. Finally, the consumption of *P. granatum* L., including the peel and edible parts, as well as its extracts, are considered safe in vitro and in vivo [103,104,105,106,107,108,109], and these results agree with the non-cytotoxicity results of *P. protopunica* Balf., however, more studies in *P. protopunica* Balf. are required to conclude that.

The data provided in this review lead us to suggest that this poorly studied species may have similar pharmacological effects like *P. granatum* L., because it belongs to the same genera and the probability that they share the same active compounds, although perhaps in different proportions. So its pharmacological properties can be better, or just the opposite.

## 4. Materials and Methods

### 4.1. Search Criteria

A search for information on the subject was made, using as inclusion criteria all the articles, books and official web pages published to date (June 2020). In this study, published peer-reviewed articles without language restrictions (unpublished data were not included) in repositories such as PubMed, ScienceDirect, Worldwide science, Springer link, Refseek, SciELO, Cochrane Library were considered as primary sources. Secondary sources of information included data from official web pages and books of taxonomic, botanical, cultivation and vulnerability information of the *P. protopunica* Balf., species with the keywords: *Punica protopunica* Balf., wild pomegranate, Socotra pomegranate, *Punica granatum* L., *Punica* gender, Lythraceae, Myrtales, *Punica protopunica* taxonomy, health effects, biological effects, composition, chemistry, cultivation, distribution and morphology.

### 4.2. Data Extraction

The data used for this article were collected, classified, summarized, analyzed, compared, discussed and written. The conclusions were made accordingly. The data extracted from each study were extracted and reported using a thematic and subtopic analysis, the results were also compared with *P. granatum* L., as a reference species.

## 5. Conclusions

In summary, this review supports the idea that the *P. protopunica* Balf. species can be considered as a powerful source of different pharmacological activities. Taking into account that it has been widely reported that this species exhibits antiviral, antimicrobial, antiprotozoal, antioxidant and anticancer activities. It was shown that there is a relationship between *P. protopunica* and *P. granatum*. It is not entirely clear whether the species remains vulnerable, but there are efforts to conserve it. The presence of various bioactive compounds could justify their effect, however, it is necessary further studies to demonstrate its pharmacological actions and their possible adverse effects. Additionally, it is essential to consider clinical studies to establish adequate doses in humans to evaluate the bioactive compounds of *P. protopunica* Balf., whereas the use of traditional medicine and complementary or alternative medicine are increasingly used in developing and developed countries.

## 6. Prospects

There is a wide range of possibilities for studying this species. In biomedical sciences, more information is needed on the effect of *P. protopunica* Balf. on human health. In vitro and in vivo models in various pathologies are necessary. It is suggested to delve into the mechanisms of action of the species in each pathology studied. Studies of the chemical composition and evaluation of its bioactive compounds (tree and especially of the fruit) are necessary, and compare these results with the composition of *P. granatum* L. Clinical studies are considered necessary to corroborate the effects reported in traditional medicine, once it has been reported that there is no toxicity. It is suggested to use other compound extraction methods and the use of high, medium and low polarity solvents.

On the other hand, the scientific community has focused all its efforts on studying *P. granatum* L. and the immense variety of cultivars it presents. There are thousands of articles on *P. granatum* L., there are high-quality books and reviews, and in most of them, they only mention *P. protopunica* Balf. as a curiosity. This review represents an effort and a call for the scientific community and the population in general to know more about this wonderful species, and to the extent possible, studies in the area of biology, health and pharmacology can be carried out. By studying the benefits of this species, a culture of respect and care can be generated. It is recommended that if scientists wish to study this species, they cultivate it in its places of origin, to preserve the species and take care of the wonderful place that houses it, Socotra.

## Figures and Tables

**Table 1:** Taxonomic positioning of *Punica protopunica* Balf.

Classification	Denomination	Common Name
Order	Myrtales	-
Family	Lythraceae	Loosestrife
Subfamily	Punicoideae	-
Genera	*Punica*	Pomegranate
Species	*Punica protopunica*	Wild pomegranate, Socotra pomegranate

Adapted from [28,29].

**Table 2:** Morphological characteristics of *P. protopunica* Balf.

*Punica protopunica* Balf.	Morphological Characteristics
**General habit**	The tree can reach a height of 2.5 to 4.5 m. It is considered a small tree or shrub, but if it reaches more than 9.14 m it can be classified as a tree, that is, it can be considered as both main forms. There are trees that are wider than they are tall (for example, trees that grow on the slopes rocks of Socotra) and trees taller than they are wide (typical of trees growing on the Socotra limestone plateau.) Generally, the tree is equal in width to height, with an upright shrub appearance.
**Bark, branches and trunk**	The bark is reddish-brown when the tree is young, but changes to a grayish hue as it grows older. The branches have thorns.
**Leaves**	The leaves grow to a length of 3 cm, in pairs on the opposite sides of the stalk, they are perennial, their most common shape is elliptical or oblong, although there are also circular or oval and obovate leaves (a single branch can have leaves of all the shapes described). Its color is dark green, with a bright tone.
**Flowers**	The flowers have obovate or oval petals, although they are sometimes heart-shaped. Its color is light pink with glitter and its shape is “trumpet”. Flowering occurs from December until the summer of the following year. Their physiology allows them to produce fertilization and pollination. It is a self-crossing species.
**Fruits**	The shape of the fruits is almost identical to that of modified tangelos (Citrus x tangelo). They retain their floral chalice tube. The peel is hard and its color when ripe is light green to greenish yellow and may or may not contain pink reflections. Inside they present a spongy pericarp with membranes (endocarp) that separate the arils into compartments. In turn, each aril contains a membrane, pulp juice and a seed.
**Seeds**	The seeds are inside the arils and there are hundreds of them, they are relatively light.

Adapted from [20,26,27,29,31].
